# Estimating Number of Nephrons in Living Pediatric Patients Using Nephrectomy Specimens

**DOI:** 10.1016/j.ekir.2024.12.027

**Published:** 2024-12-30

**Authors:** Haruhide Sakaguchi, Daishi Hirano, Yuhei Kawakami, Ai Tokunaga, Naoto Nishizaki, Amane Endo, Hiroki Miyano, Yuki Yuza, Saeko Hatanaka, Takaya Sasaki, Go Kanzaki, Nobuo Tsuboi, Kimihiko Oishi

**Affiliations:** 1 Department of Pediatrics, The Jikei University School of Medicine, Tokyo, Japan; 2 Department of Pediatrics, Kochi Medical School, Kochi University, Kochi, Japan; 3 Department of Pediatrics, Loco Clinic, Tokyo, Japan; 4 Department of Pediatrics, Juntendo University Urayasu Hospital, Urayasu, Japan; 5 Department of Pediatrics and Adolescent Medicine, Juntendo University Graduate School of Medicine, Tokyo, Japan; 6 Department of Pediatrics, Juntendo University Nerima Hospital, Tokyo, Japan; 7 Department of Hematology/Oncology, Tokyo Metropolitan Children's Medical Center, Tokyo, Japan; 8 Division of Nephrology and Hypertension, Department of Internal Medicine, The Jikei University School of Medicine, Tokyo, Japan

**Keywords:** children, glomerular filtration rate, kidney biopsy, nephron number, single nephron GFR

## Abstract

**Introduction:**

The number of nephrons is a critical determinant of renal health, because chronic kidney disease often results from a reduction in functional nephrons. Variability in the number of nephrons is influenced by genetic, racial, prenatal, and postnatal factors. Estimating the number of nephrons is crucial for establishing a baseline, before the onset of age-related nephron loss. This study aimed to estimate number of nephrons in pediatric patients by using imaging and histological techniques.

**Methods:**

This retrospective study included pediatric patients with renal tumors, who are undergoing nephrectomy and contrast-enhanced computed tomography (CT) between January 2010 and January 2021. Postsurgical renal tissue specimens were fixed, stained, and scanned into high-resolution digital images. Nonsclerotic glomerular density was calculated using stereological methods, whereas renal cortical volume was measured using ITK-SNAP software on CT images. The total number of nephrons was estimated based on cortical volume and glomerular density. Statistical analyses were conducted to identify the correlations between the number of nephrons and clinical factors.

**Results:**

Twenty-one children met the inclusion criteria. The cohort comprised 13 boys (61.9%) and 8 girls (38.1%) with a median age at nephrectomy of 2.5 years. The median number of nephrons per kidney was 925,000 (interquartile range [IQR]: 845,000–1,020,000). Higher number of nephrons was significantly correlated with older age at nephrectomy (*ρ* = 0.47, *P* = 0.036) and larger body surface area (BSA; *ρ* = 0.45, *P* = 0.034).

**Conclusion:**

This study offers new insights into pediatric nephron endowment, emphasizing the importance of early nephron assessment for predicting long-term renal outcomes.

## Graphical abstract

**Figure:**
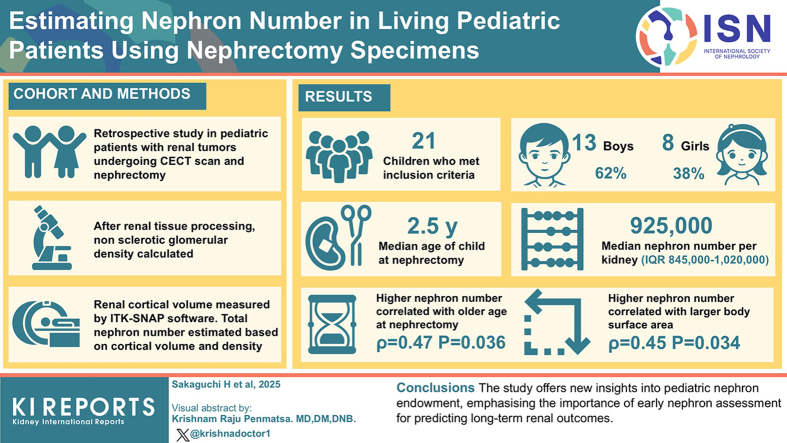

See Commentary on Page 654

The number of nephrons—the functional units of the kidney—is a critical determinant of renal health. Chronic kidney disease occurs primarily because of a reduction in the number of functional nephrons.^1^ In 1988, Brenner *et al.* proposed that reduced glomerular filtration surface area, resulting from fewer nephrons, is a risk factor for hypertension and the progression of renal disease in adults.^2^ Recent autopsy studies of individuals with healthy renal function revealed considerable interindividual variability in the number of nephrons, emphasizing the importance of differences in nephron endowment in predicting long-term renal outcomes.^3^, ^4^, ^5^, ^6^, ^7^, ^8^, ^9^, ^10^, ^11^, ^12^, ^13^

The number of nephrons are influenced by genetic, racial, prenatal, and postnatal factors, including birthweight and gestational age.^3^^,^^13^^,^^14^ Cross-sectional studies of healthy kidney donors showed an age-related decline in nonsclerosed glomeruli and increased sclerotic glomeruli, suggesting progressive nephron sclerosis and scarring with aging.^15^^,^^16^ Therefore, estimating the number of nephrons in children is crucial, because it excludes the confounding effects of age-related nephron loss and provides a baseline for future renal function assessments.

Historically, the number of nephrons were estimated exclusively through postmortem analyses,^17^ which were time-intensive, resource-demanding, and unsuitable for living subjects. Recently, a novel approach utilizing contrast-enhanced CT imaging and 3-dimensional structural analysis of pathological kidney specimens was developed to estimate total number of nephrons in living kidneys^15^ and was initially applied in adult cohorts. This study aimed to estimate the number of nephrons in pediatric patients undergoing surgical nephrectomy, utilizing the improved techniques described by Denic *et al.*^18^

## Methods

Direct informed consent was waived owing to the retrospective nature of the study, which was in accordance with the relevant ethical guidelines.^19^ We implemented a comprehensive opt-out process. Study information was publicly disseminated through our institution's website and bulletin boards, providing patients and their families the opportunity to decline participation. This opt-out approach was reviewed and approved by the Ethics Review Committee of the Jikei University School of Medicine (ID: 30-394[9415])

### Patient Selection

This study included pediatric patients with renal tumors undergoing nephrectomy at Juntendo University Hospital and Tokyo Metropolitan Children’s Medical Center, Japan, between January 1, 2010, and January 31, 2021. Inclusion criteria were nephrectomy and preoperative contrast-enhanced CT for renal tumors. The exclusion criteria were bilateral tumors, chromosomal abnormalities, or inadequate pathology specimens or CT images required for measurement.

### Definitions

Preterm birth was defined as gestational age < 37 weeks, and low birthweight was defined as birthweight < 2500 g. Proteinuria was defined as urinary protein-to-creatinine ratio ≥ 0.20 g/gCr. Hematuria was defined as the presence of ≥ 5 red blood cells per high-power field in urinary sediment on microscopic examination. The estimated glomerular filtration rate was calculated using serum creatinine (sCr) and height, based on the equation of Uemura *et al.*^20^^,^^21^

### Morphometry and Stereology of Postsurgical Renal Tissue

Postsurgical renal tissue specimens were fixed in formalin and embedded in paraffin. Two consecutive sections, each 3 μm thick, were stained with periodic acid-Schiff or hematoxylin and eosin and scanned into high-resolution digital images using an Aperio AT2 scanner (Leica Microsystems, Wetzlar, Germany). Tissue sections devoid of any artifacts that severely distorted microstructures, with at least 12 mm^2^ of the cortical area and at least 50 glomeruli, were included in the analyses. The sampling distribution of the mean approaches normality when the sample size exceeds 30, according to the central limit theorem, even in cases of nonnormal distribution.^22^ In this study, we established a more conservative minimum threshold of 50 glomeruli per sample, because this provides robust statistical power. Additional sampling beyond this threshold would not yield meaningful improvements in estimation accuracy. Subsequently, the biopsy specimens were analyzed manually by a single observer to maintain consistency in assessment.

**Figure 1:**
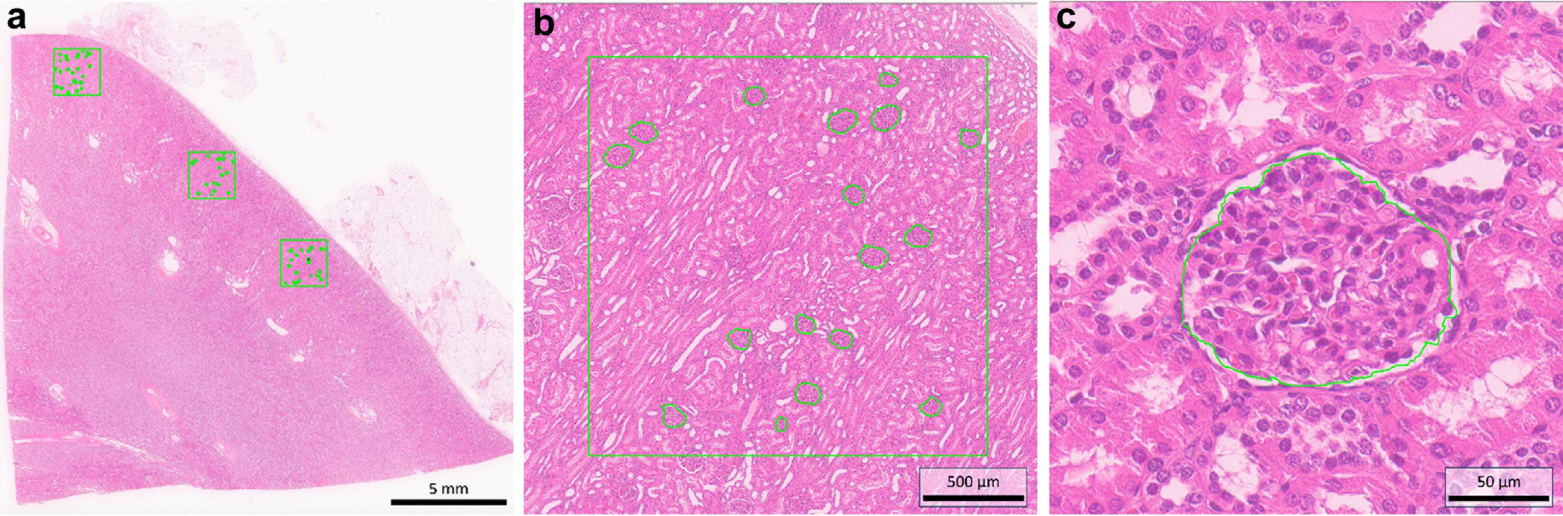
Automated stereological measurement. (a) At least 3 randomly selected squares measuring 2 × 2 mm were analyzed within the renal cortical area. (b) The number and area of glomeruli within the selected area were quantified, with at least 50 glomeruli counted, and the circles around the glomeruli were manually traced. (c) The software automatically calculated the selected glomerular area.

The density of nonsclerotic glomeruli was calculated based on stereological assessments of the cross-sectional and cortical areas identified in the biopsy specimens. Glomerular density was determined by measuring the cortical and glomerular areas. Images were evaluated using Leica Aperio Image Scope software (www.aperio.com) (Figure 1).

The mean glomerular area was calculated by dividing the total glomerular area by the total number of glomeruli in each renal tissue segment. The mean glomerular volume was estimated from the measured mean glomerular area as follows:

$$\text{Mean}\;\text{glomerular}\;\text{volume}={\left(\text{mean}\;\text{glomerular}\;\text{area}\right)}^{3/2}\times \beta /d$$

where β is a dimensionless shape coefficient (β = 1.382 for spheres) and *d* is the size distribution coefficient, which accounts for variability in glomerular size (*d* = 1.01).^23^^,^^24^

### Measurement of Renal Cortical and Parenchymal Volumes

**Figure 2:**
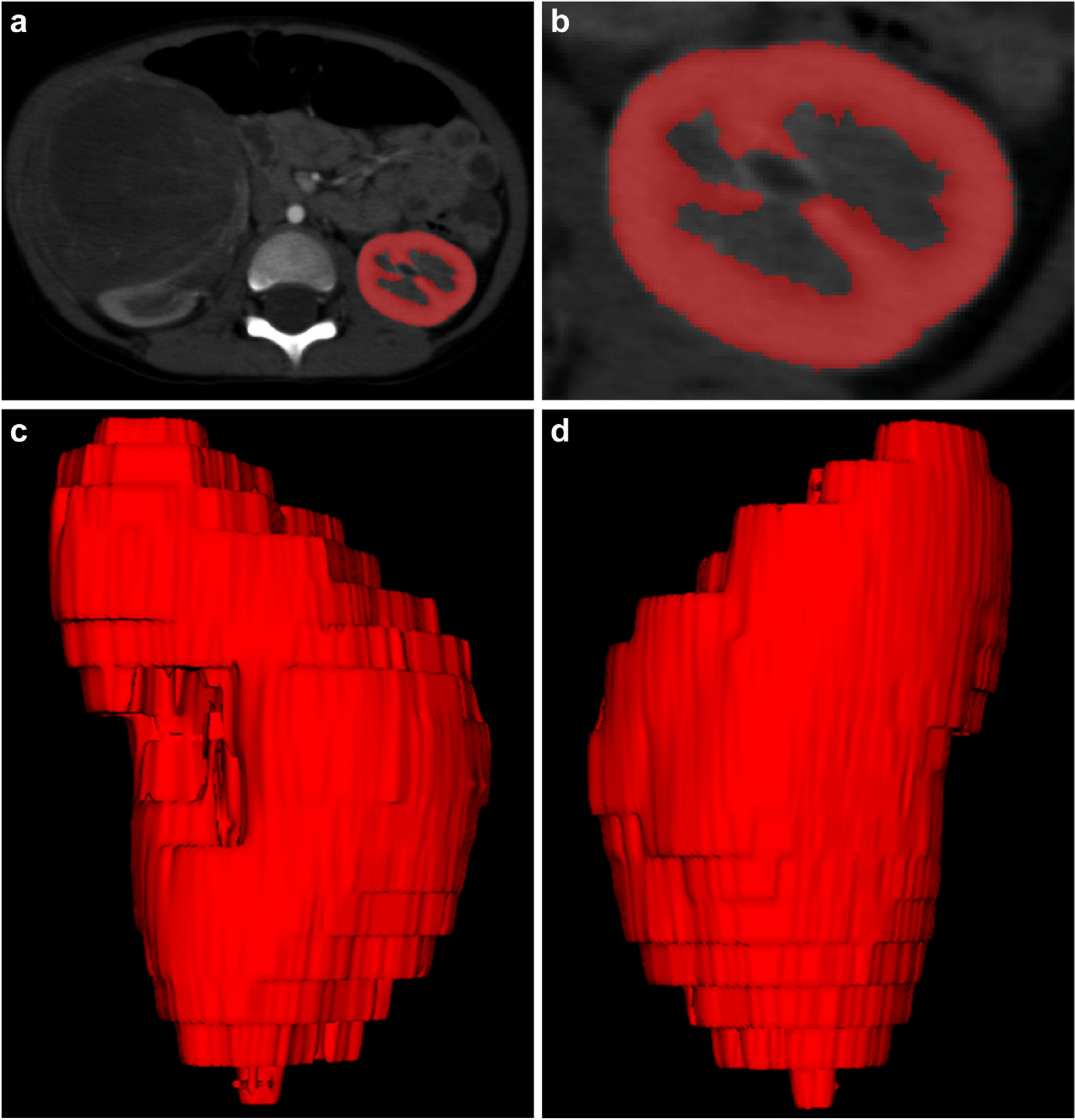
Measurement of kidney parenchymal and cortical volumes using ITK-SNAP (version 3.8.0). A subregion from the axial plane was defined manually to include both kidneys. For kidney segmentation, active contour evolution was applied to the sub-region. The cortex and medulla were defined based on intensity value thresholds. Manual correction was applied to address over- and under-segmentation errors.

Kidney parenchymal and cortical volumes were measured as described previously using ITK-SNAP version 3.8.0 (http://www.itksnap.org) to semiautomatically segment the cortex and medulla on transverse images obtained during the arterial phase on CT angiograms (Figure 2).^15^ Measurements were taken only on the unaffected kidney.

Three-dimensional cortical images were constructed semiautomatically from contrast-enhanced images obtained in the arterial phase after contrast media infusion. The renal artery, renal vein, renal pelvis, ureter, renal sinus, fat in the renal sinus, adjacent tissue, and adjacent organs were excluded from all created images.

### Estimatin of Number of Nephrons

The total number of nephrons was estimated using cortical area and glomerular density measurements, applying the modified formula developed by Denic *et al.*^18^ The calculation is expressed as:

Estimatednumberofglomeruliperkidney=(corticalvolume×glomerulardensity)/(α×β)

Where *α* (1.365) represents the correction factor for tissue perfusion loss-associated volume shrinkage (average 26.8%), and *β* (1.366) denotes the correction factor for volumetric shrinkage derived from human autopsy kidney specimens.^18^

The glomerular density (per mm^2^ of cortex) was calculated using the Weibel–Gomez stereological method as follows:^24^

$$\text{G}\text{l}\text{o}\text{m}\text{e}\text{r}\text{u}\text{l}\text{a}\text{r}\;\text{d}\text{e}\text{n}\text{s}\text{i}\text{t}\text{y}=1/\text{β}{\left(\sqrt{\text{t}\text{o}\text{t}\text{a}\text{l}}\text{n}\text{u}\text{m}\text{b}\text{e}\text{r}\;\text{o}\text{f}\;\text{g}\text{l}\text{o}\text{m}\text{e}\text{r}\text{u}\text{l}\text{i}/\text{a}\text{r}\text{e}\text{a}\;\text{o}\text{f}\;\text{c}\text{o}\text{r}\text{t}\text{e}\text{x}\right)}^{3}/\;\left(\text{t}\text{o}\text{t}\text{a}\text{l}\;\text{g}\text{l}\text{o}\text{m}\text{e}\text{r}\text{u}\text{l}\text{a}\text{r}\;\text{a}\text{r}\text{e}/\text{a}\text{r}\text{e}\text{a}\;\text{o}\text{f}\;\text{c}\text{o}\text{r}\text{t}\text{e}\text{x}\right)$$

where β is a dimensionless shape coefficient (β = 1.382 for a sphere).

### Statistical Analysis

Continuous variables are expressed as the median and IQR, whereas categorical variables are expressed as frequencies. To examine the association between separate and background factors, we used Wilcoxon's rank-sum test for continuous variables and Fisher exact test for categorical variables, as appropriate. Spearman’s correlation coefficients were calculated to assess the correlations between number of nephrons and background factors. For age-stratified analysis, a significance level of 0.05 was adjusted using the Bonferroni method to account for multiple comparisons (significance threshold: *P* < 0.017). All statistical analyses were performed using STATA (version 16.0; Stata Corp, College Station, TX), with statistical significance set at *P* < 0.05 for nonmultiple comparison analyses.

## Results

### Patient Characteristics

**Figure 3:**
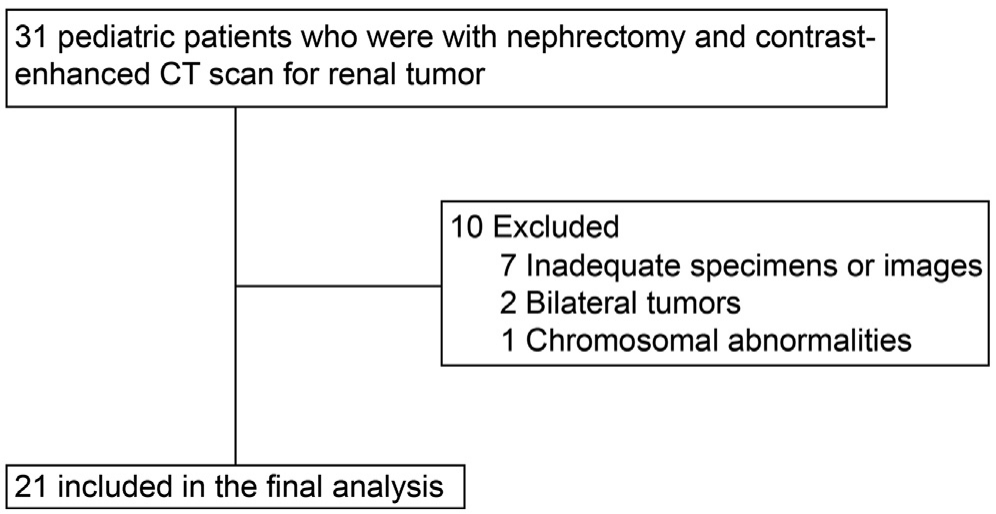
Flow diagram of participant enrolment. A total of 31 pediatric patients undergoing nephrectomy and contrast-enhanced CT for renal tumors were initially screened, and 21 were included in the study. CT, computed tomography

A total of 31 pediatric patients with renal tumors underwent nephrectomy and contrast-enhanced CT at the 2 participating institutions during the study period. Of these, 21 patients met the inclusion criteria (Figure 3). The remaining 10 patients were excluded because 5 had inadequate contrast enhancement for sufficient cortical measurement, 2 had insufficient glomeruli, 2 had bilateral renal tumors, and 1 had chromosomal abnormalities and an inadequate number of glomeruli. The characteristics of the 21 patients included in the study are summarized in Table 1. The cohort comprised 13 boys (61.9%) and 8 girls (38.1%), with a median age at nephrectomy of 2.5 years (IQR: 1.4–3.8 years). The median gestational age and birthweight were 39 weeks (IQR: 37–40 weeks) and 2804 g (IQR:2700–3280 g), respectively. The study also included 2 patients who were preterm with gestational ages of 34.1 and 36 weeks, respectively, and 2 patients with low birthweights of 2262 and 2458 g, respectively. The most common type of renal tumor was Wilms’ tumor (18 cases, 85.7%). Proteinuria or hematuria at the time of nephrectomy was observed in 1 patient each. The median sCr level was 0.28 mg/dl (IQR: 0.23–0.32 mg/dl), and that of estimated glomerular filtration rate was 109.2 ml/min per 1.73 m^2^ (IQR: 89.3–133.2 ml/min per 1.73 m^2^).

**Table 1:** Clinical characteristics of the study population at the time of nephrectomy

Patient background	Total (*n* = 21)
Boys, *n* (%)	13 (61.9)
Age (yrs), median (IQR)	2.5 (1.4–3.8)
Gestational age (wks), median (IQR)	39 (37–40)
Birthweight (g), median (IQR)	2804 (2700–3280)
Preterm birth, *n* (%)	2 (9.5)
Low birthweight, *n* (%)	2 (9.5)
Height (cm), median (IQR)	91.0 (78.0–98.3)
Body weight (kg), median (IQR)	12.9 (10.4–15.2)
Body surface area (m^2^), median (IQR)	0.571 (0.457–0.630)
Tumor type, *n* (%)	
Wilms’ tumor	18 (85.7)
Renal cell carcinoma	1 (4.8)
Clear cell renal cell sarcoma	1 (4.8)
Cystic kidney disease	1 (4.8)
Proteinuria, *n* (%)	1 (4.8)
Hematuria, *n* (%)	1 (4.8)
Serum Cr (mg/dl), median (IQR)	0.28 (0.23–0.32)
eGFR (ml/min per 1.73 m^2^ per yr), median (IQR)	109.2 (89.3–133.2)

Cr, creatinine; eGFR, estimated glomerular filtration rate; IQR, interquartile range.

### Morphometric Measurements of the Kidney

In Table 2 we present the kidney morphometric measurements. The median number of glomeruli counted per patient was 64 (IQR:60–78). The median glomerular area in this cohort was 7.05 × 10^3^ μm^2^, which is consistent with the average for this age group. The estimated number of nonsclerotic nephrons per kidney was 925,000 (IQR: 845,000–1,020,000), with a nearly 2-fold difference between the lowest and highest number of nephrons. The number of sclerotic glomeruli and degree of interstitial fibrosis were both < 1%.

**Table 2:** Morphometric measurements of the kidney

Measurement results	Total (*n* = 21)
Renal cortical volume (mm^3^/kidney), median (IQR)	69,700 (55,800–83,900)
Number of glomeruli measured (pcs), median (IQR)	64 (60–78)
Glomerular volume density (/mm^3^), median (IQR)	49.4 (38.5–55.9)
Mean glomerular area (× 10^3^ μm^2^), median (IQR)	7.05 (6.57–8.80)
Mean glomerular volume (× 10^6^ μm^3^), median (IQR)	0.81 (0.73–1.13)
Number of nephrons per kidney, median (IQR)	925,000 (845,000–1,020,000)
Total glomerular volume (× 10^3^ mm^3^), median (IQR)	0.75 (0.62–0.99)

IQR, interquartile range; pcs, pieces.

### Correlations of Number of Nephrons With Physiological Morphometric Factors

**Figure 4:**
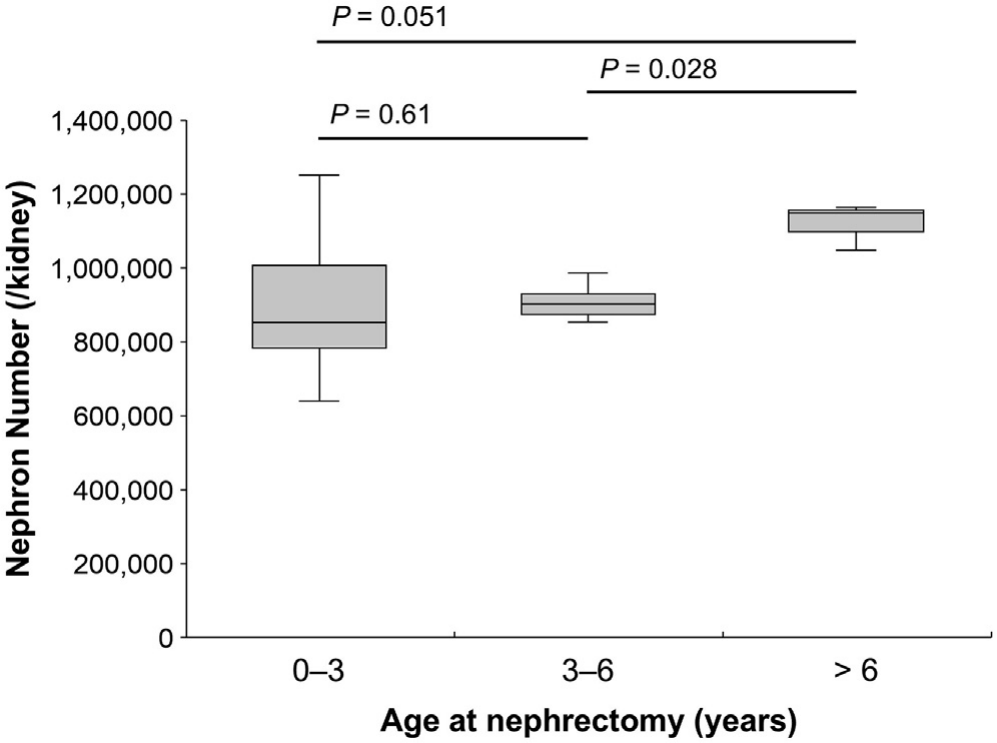
Age-stratified analysis of nephron number distribution. A box-and-whisker plot analysis was conducted to examine nephron count distributions across 3 age cohorts (0–3, 3–6, and > 6 years). Although data suggested an age-dependent upward trajectory in nephron quantity, statistical analysis revealed no significant differences among the investigated age groups.∗A basic significance level of 0.05 is corrected using the Bonferroni method to account for multiple testing (significance after correction: *P* < 0.017).

Correlation analyses were performed to investigate the relationship between number of nephrons and various physiological and clinical parameters (Table 3). The results indicated significant positive correlations between the number of nephrons and age at the time of measurement (*ρ* = 0.47), as well as BSA (*ρ* = 0.45). Subsequently, age-stratified analysis was conducted by categorizing patients into 3 distinct age groups (0–3, 3–6, and > 6 years; Figure 4). Although the data suggested an age-dependent increase in number of nephrons, the limited sample size (*n* = 21) precluded definitive statistical inference between these subgroups. Notably, neither gestational age nor birthweight exhibited significant correlations with the number of nephrons. Sex-stratified analyses were additionally performed; however, the statistical power was insufficient because of the small subgroup sample sizes, resulting in no detectable significant associations.

**Table 3:** Correlations of total number of nephrons with clinical and morphometric factors

Parameters	Spearman’s rank correction coefficient (*P-*value)		
	All (*n* = 21)	Boys (*n* = 13)	Girls (*n* = 8)
Age at nephrectomy (yrs)	0.47 (0.01^a^)	0.29 (0.34)	0.40 (0.32)
Body surface area (m^2^)	0.45 (0.02^a^)	0.36 (0.22)	0.31 (0.46)
Gestational age (wks)	−0.34 (0.19)	−0.48 (0.14)	−0.09 (0.87)
Birthweight (g)	−0.02 (0.74)	0.31 (0.33)	−0.18 (0.70)
Body weight (kg)	0.28 (0.22)	0.34 (0.26)	0.26 (0.53)
Serum Cr (mg/dL)	0.38 (0.10)	0.41 (0.17)	0.13 (0.12)
eGFR (ml/min per 1.73 m^2^ per yr)	0.10 (0.62)	−0.26 (0.39)	−0.24 (0.57)
Glomerular volume density (/mm^3^)	0.10 (0.67)	0.13 (0.68)	0.12 (0.78)
Mean glomerular area (μm^2^)	−0.08 (0.72)	−0.14 (0.64)	−0.17 (0.69)
Mean glomerular volume (μm^3^)	−0.08 (0.72)	−0.14 (0.64)	−0.17 (0.69)

Cr, creatinine; eGFR, estimated glomerular filtration rate.

a Statistically significance (**P** < 0.05).

## Discussion

This study aimed to estimate the number of nephrons in the kidneys of living children with renal tumors. The median number of nephrons per kidney was 925,000, with variation observed across the cohort. Older age at nephrectomy and larger BSA were correlated with higher number of nephrons in children. These findings provide novel insights into nephron endowment in pediatric populations, enhancing our understanding of renal health and disease. To the best of our knowledge, this is the first study to estimate the number of nephrons in the kidneys of living children, making a substantial advancement over previous studies, which primarily focused on adult populations. The use of contrast-enhanced CT and high-resolution digital imaging enabled accurate and precise morphometric analyses. The large number of cortical areas and glomeruli measured in each patient minimized sampling bias and enhanced the reliability of our results. This methodological strength distinguished our study from previous studies relying on postmortem specimens or renal biopsies with limited glomerular counts.^15^^,^^25^

In this study, the estimated number of nephrons per kidney in children with a median age of 2.5 years was approximately 925,000. Previous studies have primarily focused on adult populations and reported considerable interindividual variability in number of nephrons (Table 4).^5^^,^^12^^,^^16^^,^^25^, ^26^, ^27^ For example, Sasaki *et al.* reported a mean total number of nephrons of 710,000 in a Japanese cohort of kidney transplant donors with a median age of 56.7 years^25^ and Kanzaki *et al.* reported approximately 670,000 nephrons in Japanese adults.^12^ Both studies estimated fewer nephrons compared with our findings, suggesting that nephrons gradually undergo sclerosis with age, culminating in eventual scarring. A cross-sectional study involving a large cohort of healthy kidney transplant donors confirmed a decrease in nonsclerosed glomeruli and an increase in globally sclerosed glomeruli with age.^15^^,^^16^ The total number of nonsclerosed glomeruli declined at a rate of 6200 per year in this cohort.^15^^,^^16^ Patients aged ≥ 70 years had lost approximately 345,000 glomeruli per kidney, which is approximately 2.5 times greater than the number of sclerotic glomeruli detected in this age group.^15^^,^^16^ Thus, estimating number of nephrons in children is crucial, because it helps exclude the confounding effects of age-related nephron loss.

**Table 4:** Comparison of findings of the present study with previous reports

Study	Sample	Age	*n*	Nephron number (/kidney)		Reference
				Mean	Range	
USA (African American)	kidney autopsy	Adult	176	898,748	210,332–2,702,079	26
USA (White)	kidney autopsy	Adult	132	894,119	227,327–1,660,232	26
Australia (Aboriginal)	kidney autopsy	Adult	19	713,209	364,161–1,129,233	26
Australia (Anglo-Celtic)	kidney autopsy	Adult	24	861,541	380,517–1,493,665	26
Senegal	kidney autopsy	Adult	47	988,263	536,171–1,764,421	26
Germany	kidney autopsy	Adult	20	1,074,414	531,140–1,959,914	5
France	kidney autopsy	Adult	28	1,107,000	655,000–1,554,000	27
Japan	kidney autopsy	Adult	9	666,140	-	12
USA	living kidney	Adult	1638	915,131	-	15, 16
Japan	living kidney	Adult	44	710,000	290,000–1,340,000	25
USA	living kidney	Adult	3020	1,170,000	-	18
USA	living kidney with a tumor	Adult	1354	990,000	-	18
Present study	living kidney	Infant	21	925,000^a^	654,000–1,251,000	

a Median.

In this study, no significant correlations were found between number of nephrons and gestational age or birthweight. However, significant positive correlations were observed between number of nephrons and age at nephrectomy, BSA, and renal cortical volume. Among the surrogate markers identified to date, birthweight has been shown to be closely correlated with total number of nephrons.^4^^,^^6^^,^^7^^,^^9^ Nephron formation in humans occurs between approximately 5 and 36 weeks of gestation, with no new nephrons formed postnatally. Approximately 60% of nephrons are formed during the late stages of pregnancy^28^; preterm and low birthweight infants are considered congenitally predisposed to having a lower number of nephrons. Autopsy studies in adults have shown a positive correlation between birthweight and the number of glomeruli, with an increase of approximately 250,000 glomeruli for each 1 kg increase in birthweight.^4^ However, this study found no significant correlations between gestational age, birthweight, and number of nephrons. This may have been attributable to the small numbers of preterm infants (*n* = 2, both ≥ 34 weeks of gestation) and low birthweight infants (*n* = 2, both ≥ 2200 g), resulting in limited variability in these parameters. In addition, in the Japanese study by Kanzaki *et al.* and Sasaki *et al.*,^12^^,^^25^ participants were Japanese and adults, none were pediatric, and no reference to gestational age or birthweight was provided.

Notably, significant positive correlations were observed between number of nephrons, age at nephrectomy, and BSA. Previous studies reported that the number of nephrons are lower in Japanese adults than in Western populations,^12^ whereas similar number of nephrons have been observed in Australian Aboriginal and Japanese populations.^7^ These differences can be attributed to smaller body size and corresponding kidney size, as well as genetic factors, in Japanese and Aboriginal populations relative to Western populations. These factors likely explain the significant correlations observed between number of nephrons and body size in this study. Regarding the relationship between age at measurement and number of nephrons, number of nephrons generally remain constant during childhood. The observed correlation between number of nephrons and age may be influenced by compensatory hypertrophy of the contralateral kidney. In general, in patients with functional heminephrosis, particularly those maintaining normal renal function, compensatory enlargement of the remaining kidney is well-documented.^29^^,^^30^ Previous studies on children with multicystic dysplastic kidneys demonstrated that contralateral hypertrophy occurs at a mean of 2.7 years postdiagnosis, with 90% exhibiting compensatory changes by age of 10 years.^31^ The magnitude of this compensatory effect likely intensifies with disease duration, which inherently increases with patient age.

This study had some limitations. First, the cohort included patients with renal tumors, predominantly Wilms’ tumors, which arise from metanephric blastema and may affect nephron progenitors, potentially influencing nephron counts.^32^ Although our findings showed normal glomeruli and renal stroma, caution is warranted when extrapolating these results to healthy kidneys. Second, the slice thickness of the CT images was not known owing to the multicenter and retrospective nature of the study. Whereas Denic e*t al.* implemented cortical volume corrections using a multivariate linear regression model based on CT slice thickness,^18^ the heterogeneity of our imaging data precluded the application of such methodological refinements. This limitation should be considered when interpreting our volumetric analyses. Third, not all glomeruli in the pathological specimens were analyzed. Nephrons in which the glomeruli are located in the superficial cortex are shorter than those in the juxtamedullary cortex; moreover, glomeruli in the juxtamedullary cortex are generally larger than those in the superficial cortex.^33^ Finally, the retrospective nature of the study and the specific patient population limited the generalizability of our findings.

To our knowledge, this is the first study to estimate number of nephrons in the kidneys of living children. The estimated number of nephrons were higher than those previously reported in adults, reflecting the age-related decline in nephron endowment. These findings provide a valuable reference for pediatric nephrology and underscore the importance of early and accurate assessment of number of nephrons. Further research is needed to elucidate the factors influencing nephron endowment and their implications on renal health across different populations.

## Disclosure

All the authors declared no competing interests.
